# Usefulness of the heart rate variability test in predicting intradialytic hypotension in patients undergoing chronic haemodialysis

**DOI:** 10.1093/ckj/sfae102

**Published:** 2024-04-08

**Authors:** Yohan Park, Ji Won Lee, Se-Hee Yoon, Won Min Hwang, Sung-Ro Yun, Ji-Young Son, Byung Ha Chung, Jiwon Min

**Affiliations:** Division of Nephrology, Department of Internal Medicine, Konyang University Hospital, College of Medicine, Konyang University, Daejeon, Republic of Korea; Division of Nephrology, Department of Internal Medicine, Konyang University Hospital, College of Medicine, Konyang University, Daejeon, Republic of Korea; Division of Nephrology, Department of Internal Medicine, Konyang University Hospital, College of Medicine, Konyang University, Daejeon, Republic of Korea; Division of Nephrology, Department of Internal Medicine, Konyang University Hospital, College of Medicine, Konyang University, Daejeon, Republic of Korea; Division of Nephrology, Department of Internal Medicine, Konyang University Hospital, College of Medicine, Konyang University, Daejeon, Republic of Korea; Division of Nephrology, Department of Internal Medicine, Bucheon St. Mary's Hospital, College of Medicine, Catholic University of Korea, Seoul, Republic of Korea; Division of Nephrology, Department of Internal Medicine, Seoul St. Mary's Hospital, College of Medicine, Catholic University of Korea, Seoul, Republic of Korea; Division of Nephrology, Department of Internal Medicine, Bucheon St. Mary's Hospital, College of Medicine, Catholic University of Korea, Seoul, Republic of Korea

**Keywords:** autonomic nervous function, haemodialysis, heart rate variability, intradialytic hypotension, nadir 90 criterion

## Abstract

**Background:**

Intradialytic hypotension (IDH) is the primary complication of haemodialysis (HD); however, its diverse pathophysiology and inconsistent definitions complicate its prediction. Despite attempts using the heart rate variability (HRV) test for IDH prediction, studies on its usefulness for predicting IDH diagnosed per the nadir 90 criterion are lacking. We aimed to evaluate HRV test efficacy and reproducibility in predicting IDH based on the nadir 90 criterion.

**Methods:**

Seventy patients undergoing HD participated in this multicentre prospective observational study. The HRV test was performed during non-HD periods and IDH was monitored during 12 HD sessions. IDH was diagnosed according to the nadir 90 criterion, defined as a decrease in systolic blood pressure of ≤90 mmHg during HD. After monitoring, the HRV test was repeated. An HRV–IDH index was developed using multivariate logistic regression analysis employing HRV test parameters. The predictive power of the HRV–IDH index was analysed using the area under the receiver operating characteristics curve (AUROC). Reproducibility was evaluated using correlation analysis of two HRV tests on the same patient.

**Results:**

There were 37 and 33 patients in the IDH and non-IDH groups, respectively. The HRV–IDH index predicted IDH occurrence with AUROCs of 0.776 and 0.803 for patients who had experienced at least one or repeated IDH episodes, respectively. Spearman's correlation coefficient for HRV–IDH indices was 0.859 for the first and second HRV tests.

**Conclusions:**

The HRV test holds promise for predicting IDH, particularly for patients with recurring IDH diagnosed based on the nadir 90 criterion.

## Graphical Abstract

**Graphical Abstract:**
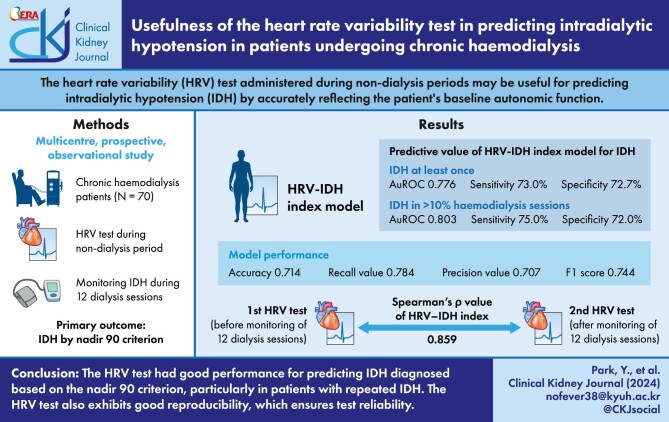

KEY LEARNING POINTS **What was known:**

- The heart rate variability (HRV) test is a non-invasive and simple test for assessing autonomic function.
- The HRV test is useful for predicting intradialytic hypotension (IDH) in patients undergoing haemodialysis.
- The efficacy of the HRV test for predicting IDH diagnosed based on the nadir 90 criterion has not been assessed.

**This study adds:**

- The HRV test was performed during non-dialysis periods on eligible patients to accurately evaluate baseline autonomic function.
- The HRV test displayed robust performance for predicting IDH diagnosed based on the nadir 90 criterion.
- The results of HRV tests performed twice on the same patient were strongly correlated, suggesting good reproducibility.

**Potential impact:**

- Assessing baseline autonomic function through the HRV test can aid in the identification of haemodialysis patients with autonomic dysfunction.
- In the future, accurate evaluation of baseline autonomic function using HRV will greatly contribute to the management of IDH.

## INTRODUCTION

Intradialytic hypotension (IDH) is the primary complication of haemodialysis (HD), occurring in 10–40% of dialysis sessions. IDH causes discomfort for patients during HD and is associated with vascular access failure, cardiovascular events, cognitive function impairment and increased all-cause mortality [1–5]. Despite recent advances in HD, the varying pathophysiology of IDH presents challenges in predicting and preventing IDH [6–9]. Furthermore, the reported incidence of IDH varies because studies use different definitions for diagnosis [8]. The Kidney Disease Outcomes Quality Initiative (KDOQI) guideline defines IDH as a decrease in systolic blood pressure (SBP) of ≥20 mmHg or a decrease in mean arterial pressure of ≥10 mmHg, accompanied by hypotensive symptoms [10]. The UK Renal Association guideline defines IDH as any symptomatic decrease in BP that requires immediate intervention [11]. However, symptoms are subjective indicators and may be missed. Therefore, several studies define IDH based solely on BP [12–14]. A recent study identified the nadir 90 criterion as being most closely associated with patient mortality [15].

IDH pathophysiology includes autonomic dysfunction [16], which occurs in patients with end-stage kidney disease owing to repeated exposure to uraemia [17, 18]. The heart rate variability (HRV) test indirectly evaluates autonomic nervous function (ANF) by analysing small differences in the intervals between heartbeats [19, 20]. Several studies have attempted to predict IDH by evaluating ANF using the HRV test. However, in these studies, IDH was not defined by the nadir 90 criterion. Furthermore, HD itself can affect autonomic nervous status; this might have significantly confounded the results of studies in which HRV tests were conducted during HD sessions [21–24].

We hypothesized that underlying autonomic function status assessment would help predict IDH. HRV tests were conducted during non-HD periods to evaluate ANF with greater accuracy. We also evaluated HRV test usefulness for predicting IDH, diagnosed per the nadir 90 criterion.

## MATERIALS AND METHODS

### Study population

This multicentre, prospective, observational cohort study recruited patients undergoing HD at Konyang University Hospital and Bucheon St. Mary's Hospital between October 2021 and December 2022. The inclusion criteria were adults >18 years of age undergoing regular 4-h HD sessions three times a week. The exclusion criteria were maintenance HD vintage of <90 days, arrhythmia (atrial fibrillation or flutter) affecting HRV test results, pre-dialysis SBP <90 mmHg, hospitalization for acute illness within the previous 1 month, terminal illness with insufficient life expectancy and low compliance due to psychiatric illness.

This study complied with the Declaration of Helsinki and was approved by the Institutional Review Boards of Konyang University Hospital (KYUH 2021-08-004-012) and Bucheon St. Mary's Hospital (HC22OISI0023). All patients provided written informed consent to participate.

### HRV test and IDH monitoring

**Figure 1:**
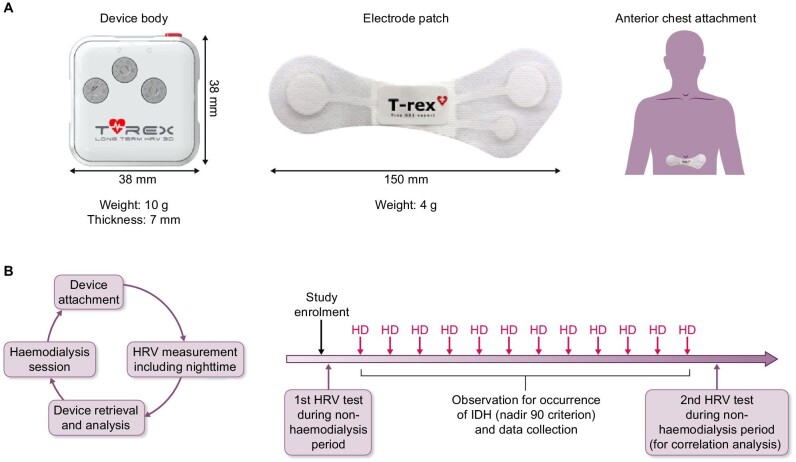
Device for HRV analysis and study protocol. **(A)** T-REX portable electrocardiogram monitoring device. The body of the device is assembled onto the electrode patch and attached to the anterior chest for measurement. The total weight of the device is only 14 g, with dimensions measuring 150 mm in width, 38 mm in length and 7 mm in thickness. The device causes no discomfort while wearing it. **(B)** Study protocol. After the HD session, the device was attached to the patient. HRV was measured for up to 34 h, including at night-time (00:00–04:00 a.m.). The device was retrieved at the visit for the next HD session. Twelve HD sessions were monitored thereafter. At the end of the monitoring period, the second HRV test was performed in the same manner.

HRV was measured using T-REX (Taewoong Medical, Seoul, Republic of Korea), a portable electrocardiogram device developed for HRV analysis. T-REX is a small, lightweight device that is attached to the anterior chest during measurement. The device causes no discomfort, can collect data for up to 34 h and is unaffected by motion artefacts (Fig. 1A) [25].

To measure HRV during non-HD periods, each patient was discharged after the HD session with the T-REX device attached. The device was retrieved at the start of the next HD session for data collection. IDH occurrence was monitored during 12 subsequent HD sessions. IDH was diagnosed according to the nadir 90 criterion, defined as a decrease in SBP to ≤90 mmHg during HD or a decrease in SBP to ≤100 mmHg when pre-HD SBP was ≥160 mmHg. If no specific symptoms were reported during HD, vital signs were measured hourly. In case of reported symptoms, the nursing staff promptly checked the vital signs. After monitoring during the 12 HD sessions, HRV measurements were repeated using the T-REX device (Fig. 1B).

### Clinical, laboratory and dialysis data collection

Clinical data, including age, sex, body mass index (BMI), comorbidities and medication history, were collected at baseline. Laboratory data, including complete blood counts [white blood cell (WBC) count, haemoglobin level, haematocrit level and platelet count], serum chemistry (total protein, albumin and glucose levels), electrolytes [sodium (Na), potassium, chloride, calcium (Ca) and inorganic phosphorus] and intact parathyroid hormone levels, were retrieved from the most recent analyses within 1 month before study enrolment. Dialysis data were collected as the averaged results of the 12 HD sessions and included whether haemodiafiltration was performed, dialysis vintage, Kt/V, urea reduction ratio, dialysate temperature, dialysate Na and Ca concentrations, dialyser surface area, ultrafiltration rate, blood flow rate and pre- and post-dialysis BP.

### Body composition measurement (BCM), transthoracic echocardiography (TTE) and cardiothoracic ratio data collection

BCM data, including body weight, overhydration, total body water, extracellular and intracellular water content and oedema index [calculated as extracellular water content/total body water content (%)], were retrieved from the most recent analyses within 3 months before study enrolment. The Body Composition Monitor (Fresenius Medical Care, Bad Homburg, Germany) device was used following the manufacturer's instructions [26].

TTE data were retrieved from the most recent results within 3 months before study enrolment and included ejection fraction, early:atrial filling velocity ratio (E:A ratio), septal diastolic mitral annulus velocity (e′ velocity), lateral e′ velocity, early diastolic mitral inflow velocity:early diastolic mitral annulus velocity ratio (E:e′ ratio), left ventricular (LV) diastolic dimension, left atrial diastolic dimension, intraventricular septal dimension, LV posterior wall dimension, maximal tricuspid regurgitation velocity, right ventricular systolic pressure, presence of regional wall motion abnormality and presence of inferior vena cava plethora. All TTE procedures were performed by cardiologists. Cardiothoracic ratio data were collected from post-dialysis simple chest radiography performed within 1 month before study enrolment.

### Analysis of HRV test results

R peaks were automatically detected and analysed using T-REX integrated software. R–R intervals (RRIs) were calculated and ectopic RRIs (differing from preceding beats by >20%) were excluded from the analysis. HRV parameters were calculated according to the guidelines of the task forces of the European Society of Cardiology and North American Society of Pacing and Electrophysiology [19]. The mean heart rate, standard deviation of the N–N interval (SDNN), root mean square difference (RMSSD) of successive N–N intervals, number of pairs of adjacent N–N intervals differing by >50 ms (NN50) and NN50 count divided by the total number of all N–N intervals (pNN50) were measured as time-domain parameters. Spectral analysis was performed using a Welch periodogram with 4-Hz resampling with linear interpolation, 64-s windows and 75% overlap [27]. Very low frequency (VLF; 0.003–0.04 Hz), low frequency (LF; 0.04–0.15 Hz), high frequency (HF; 0.15–0.40 Hz), total power (TP) and the LF:HF ratio were measured using spectral analysis. All HRV data were calculated at 5-min intervals. As daytime activity levels can affect autonomic nervous status, the average values of data collected between 00:00 and 04:00 a.m. were used.

### Statistical analyses

Continuous variables are expressed as mean ± standard deviation (SD) and were compared using Student's *t*-test. Categorical variables are expressed as counts and percentages and were analysed using chi-squared or Fisher's exact tests. Multivariate logistic regression analysis was used to develop an HRV–IDH index using the collected HRV parameters, including SDNN, RMSSD, NN50, pNN50, TP, VLF, LF, HF and LF:HF ratio. To analyse the predictive power of the HRV–IDH index, receiver operating characteristics (ROC) curve analysis was performed and the cut-off value was set based on the value with the highest sensitivity and specificity [28]. Multivariate logistic regression analysis was used to evaluate whether autonomic dysfunction based on the cut-off HRV–IDH index value is an independent risk factor for IDH. Referring to the model with the best prediction of IDH occurrence in a previous study and assuming that it has a medium–large effect size (*f*^2^ = 0.25), the target number of study participants was 80 to secure statistical reliability with α = 0.05 and β = 0.80. The statistical power was calculated using a post hoc power analysis. To evaluate the reproducibility of the HRV test results, Spearman's correlation analysis was used to analyse correlations between the parameters of the first and second HRV tests. *P*-values <.05 indicated statistical significance. All statistical analyses were performed using SPSS version 24 (IBM, Armonk, NY, USA) and G*power 3.1.9.7 (Heinrich-Heine-Universität Düsseldorf, Düsseldorf, Germany).

## RESULTS

### Comparison of baseline characteristics and incidence of IDH

Of the 81 recruited patients, 3 with atrial fibrillation and 5 with a HD vintage of <90 days were excluded. Three patients were lost to follow-up. The complete data of 70 patients were analysed (Supplemental Figure S1). Of these, 37 patients experienced IDH at least once, while 33 did not. Table 1 summarizes the baseline characteristics of the IDH and non-IDH groups. The IDH group included significantly more females, patients with diabetes mellitus (DM) and fewer patients with hypertension than the non-IDH group. Further, the IDH group had a significantly lower dialyser surface area and blood flow rate. Pre-dialysis SBP and diastolic BP (DBP) did not differ between the two groups, whereas the IDH group exhibited significantly lower post-dialysis SBP and DBP than the non-IDH group. The WBC count was significantly greater in the IDH group. The IDH group used antihypertensive medication significantly less frequently and antihypotensive medication (midodrine) significantly more frequently than the non-IDH group.


\begin{eqnarray*}
\text {HRV-IDH index} = \displaystyle\frac{{{{e}^{\left( {1.7394 + (0.1850) \times (NN50) + ( - 0.0344) \times (TP) + (0.0359) \times (VLF) + (0.0341) \times (LF) + ( - 0.2301) \times \left( {\frac{{LF}}{{HF}}\textit{ratio}} \right)} \right)}}}}{{1 + {{e}^{\left( {1.7394 + (0.1850) \times (NN50) + ( - 0.0344) \times (TP) + (0.0359) \times (VLF) + (0.0341) \times (LF) + ( - 0.2301) \times \left( {\frac{{LF}}{{HF}}\textit{ratio}} \right)} \right)}}}},
\end{eqnarray*}

where *e* is Euler's constant.

**Table 1:** Comparison of baseline characteristics between the IDH and non-IDH groups.

Characteristics	Total (*n* = 70)	Non-IDH (*n* = 33)	IDH (*n* = 37)	*P*-value
Age ≥65 years, *n* (%)	31 (44.3)	16 (48.5)	15 (40.5)	.504
Female, *n* (%)	35 (50.0)	12 (36.4)	23 (62.2)	.031
Height (cm)	160.7 ± 8.5	162.0 ± 8.8	159.6 ± 8.1	.239
Body surface area (m^2^)^a^	1.62 ± 0.17	1.64 ± 0.18	1.61 ± 0.17	.568
BMI (kg/m^2^)	23.2 ± 3.7	22.9 ± 3.4	23.5 ± 3.9	.544
Comorbidities, *n* (%)				
DM	45 (64.3)	15 (45.5)	30 (81.1)	.002
HTN	54 (77.1)	29 (87.9)	25 (67.6)	.043
CHF	10 (14.3)	4 (12.1)	6 (16.2)	.739
CAD	8 (11.4)	4 (12.1)	4 (10.8)	1.000
Cerebrovascular diseases	7 (10.0)	3 (9.1)	4 (10.8)	1.000
PAOD	8 (11.4)	2 (6.1)	6 (16.2)	.266
Dialysis information				
Hemodiafiltration, *n* (%)	16 (22.9)	10 (30.3)	6 (16.2)	.161
Dialysis vintage (months)	68.6 ± 67.6	52.3 ± 51.7	78.7 ± 78.4	.178
KT/V	1.77 ± 0.31	1.74 ± 0.30	1.80 ± 0.32	.480
URR (%)	76.4 ± 6.0	76.1 ± 6.3	76.8 ± 5.7	.611
Dialysate temperature (°C)	36.48 ± 0.05	36.46 ± 0.06	36.49 ± 0.03	.085
Dialysate Na (mEq/l)	139.2 ± 1.6	139.2 ± 1.5	139.2 ± 1.8	.874
Dialysate Ca (mg/dl)	6.03 ± 0.22	6.07 ± 0.05	6.00 ± 0.29	.119
Dialyser surface area (m^2^)	1.67 ± 0.21	1.74 ± 0.19	1.60 ± 0.20	.007
UF rate (kg/4 h)	2.3 ± 0.7	2.2 ± 0.8	2.3 ± 0.6	.638
Blood flow rate (ml/min)	270.8 ± 16.2	277.2 ± 14.2	265.1 ± 15.8	.001
Pre-dialysis SBP (mmHg)	148.2 ± 22.2	145.9 ± 19.3	150.3 ± 24.7	.418
Pre-dialysis DBP (mmHg)	71.9 ± 13.0	73.2 ± 13.4	70.7 ± 12.6	.440
Post-dialysis SBP (mmHg)	130.9 ± 20.4	142.6 ± 17.7	120.5 ± 16.8	<.001
Post-dialysis DBP (mmHg)	69.2 ± 11.7	75.3 ± 11.9	63.8 ± 8.4	<.001
Laboratory findings				
WBC (count/μl)	6.32 ± 2.38	5.34 ± 1.64	7.20 ± 2.60	.001
Hb (g/dl)	11.2 ± 1.2	10.9 ± 1.2	11.4 ± 1.2	.128
Hct (%)	34.1 ± 3.9	33.2 ± 4.0	34.8 ± 3.7	.082
PLT (×1000/μl)	177.8 ± 71.0	176.5 ± 68.1	178.9 ± 74.3	.890
Protein (g/dl)	6.72 ± 0.47	6.65 ± 0.40	6.79 ± 0.53	.217
Albumin (g/dl)	3.88 ± 0.46	3.91 ± 0.38	3.85 ± 0.52	.585
Glucose (mg/dl)	145.0 ± 57.1	144.8 ± 58.2	145.1 ± 57.0	.985
Na (mEq/l)	136.1 ± 3.2	136.5 ± 3.1	135.6 ± 3.2	.238
K (mEq/l)	4.78 ± 0.73	4.77 ± 0.69	4.78 ± 0.78	.927
Cl (mEq/l)	99.3 ± 4.1	100.2 ± 3.8	98.5 ± 4.3	.094
Ca (mg/dl)	8.93 ± 1.83	9.10 ± 2.56	8.77 ± 0.73	.455
Inorganic P (mg/dl)	4.64 ± 1.67	4.37 ± 1.66	4.87 ± 1.67	.213
Intact PTH (pg/ml)	175.0 ± 124.0	173.3 ± 100.9	176.6 ± 142.9	.911
Medication, *n* (%)				
RASI	28 (40.0)	17 (51.5)	11 (29.7)	.063
Beta-blocker	13 (18.6)	8 (24.2)	5 (13.5)	.249
CCB	27 (38.6)	18 (54.5)	9 (24.3)	.010
Furosemide	14 (20.0)	9 (27.3)	5 (13.5)	.151
Insulin	19 (27.1)	8 (24.2)	11 (29.7)	.606
Sulfonylurea	20 (28.6)	9 (27.3)	11 (29.7)	.820
DPP-IV inhibitor	27 (38.6)	8 (24.2)	19 (51.4)	.020
TZD	10 (14.3)	6 (18.2)	4 (10.8)	.499
Midodrine	10 (14.3)	0 (0)	10 (27.0)	.001
Darbepoietin	45 (64.3)	24 (72.7)	21 (56.8)	.164
Darbepoietin dose (μg)	56.4 ± 45.6	66.3 ± 53.7	45.2 ± 31.7	.113

Values are presented as mean ± SD unless stated otherwise.

a Body surface area was calculated using the Du Bois method.

BMI: body mass index; DPP-IV: dipeptidyl peptidase-4 inhibitor; PAOD: peripheral arterial occlusive disease; PLT: platelet; PTH: parathyroid hormone; TZD: thiazolidinedione; UF: ultrafiltration; URR: urea reduction ratio.

Overall, 840 HD sessions were examined, with IDH occurring in 106 (12.6%) sessions. Thirty-seven (52.9%) patients experienced IDH at least once and 20 (28.6%) repeatedly experienced IDH in >10% of the HD sessions (Supplemental Table S1).

### Comparison of the BCM, TTE, cardiothoracic ratio and HRV test results

The BCM results revealed significantly lower total body, extracellular and intracellular water content in the IDH group, whereas there was no significant difference in oedema index between the two groups. The TTE results revealed no significant intergroup differences in any parameter, including ejection fraction. Additionally, there was no significant intergroup difference in the cardiothoracic ratios (Table 2).

**Table 2:** Comparison of BCM, TTE results and cardiothoracic ratio between the IDH and non-IDH groups.

Characteristics	Non-IDH (*n* = 33)	IDH (*n* = 37)	*P*-value
Body composition measurements			
Weight (kg)	61.9 ± 12.0	61.5 ± 11.2	.907
Overhydration (l)	1.30 ± 2.32	0.90 ± 2.30	.480
Total body water (l)	35.8 ± 8.9	31.3 ± 7.3	.023
Extracellular water (l)	16.3 ± 4.0	14.5 ± 3.3	.046
Intracellular water (l)	19.5 ± 5.3	16.8 ± 4.6	.021
Oedema index	0.46 ± 0.04	0.47 ± 0.04	.386
Echocardiography			
Ejection fraction (%)	65.3 ± 9.3	62.8 ± 9.9	.280
E:A ratio	0.81 ± 0.44	0.88 ± 0.55	.569
Septal e′ (cm/s)	0.79 ± 2.23	0.92 ± 2.26	.823
Lateral e′ (cm/s)	1.52 ± 3.14	1.61 ± 2.97	.904
E:e′ ratio	12.98 ± 6.24	12.87 ± 6.48	.946
LV diastolic dimension (cm)	6.30 ± 3.52	5.47 ± 2.09	.232
LA diastolic dimension (cm)	3.94 ± 2.00	3.58 ± 1.27	.369
IV septal dimension (cm)	1.01 ± 0.21	0.99 ± 0.16	.651
LV posterior wall dimension (cm)	0.96 ± 0.16	0.95 ± 0.14	.697
Maximum TR velocity (m/s)	2.41 ± 0.56	2.28 ± 0.70	.450
RV systolic pressure (mmHg)	29.0 ± 10.7	27.7 ± 15.9	.728
Presence of RWMA, *n* (%)	5 (15.2)	5 (13.5)	1.000
Presence of IVC plethora, *n* (%)	0 (0)	1 (2.7)	1.000
Cardiothoracic ratio (%)	50.7 ± 7.4	48.6 ± 6.8	.223

Values are presented as mean ± SD.

IV: interventricular; IVC: inferior vena cava; LA: left atrial; LV: left ventricular; RV: right ventricular; RWMA: regional wall motion abnormality; TR: tricuspid regurgitation.

The HRV test results of the groups are summarized in Table 3. Both VLF and HF were significantly lower in the IDH than the non-IDH group. Additionally, the SDNN, TP and LF:HF ratio were also lower in the IDH group.

**Table 3:** Comparison of HRV test results between the IDH and non-IDH groups.

Test	Non-IDH (*n* = 33)	IDH (*n* = 37)	*P*-value
SDNN (ms)	27.7 ± 12.3	21.9 ± 12.5	.053
RMSSD (ms)	14.6 ± 10.4	14.2 ± 12.9	.880
NN50 count	6.46 ± 17.46	11.75 ± 28.66	.361
pNN50 (%)	2.48 ± 7.00	3.67 ± 9.07	.545
Total power (ms^2^)	527.8 ± 432.2	326.7 ± 388.7	.070
VLF (ms^2^)	284.5 ± 253.0	174.1 ± 248.7	.020
LF (ms^2^)	163.0 ± 165.4	81.9 ± 107.3	.318
HF (ms^2^)	64.8 ± 91.6	45.4 ± 69.5	.044
LF/HF ratio	5.35 ± 4.48	3.50 ± 3.47	.056

Values are presented as mean ± SD.

### Development of the HRV–IDH index and predictive power for IDH

**Figure 2:**
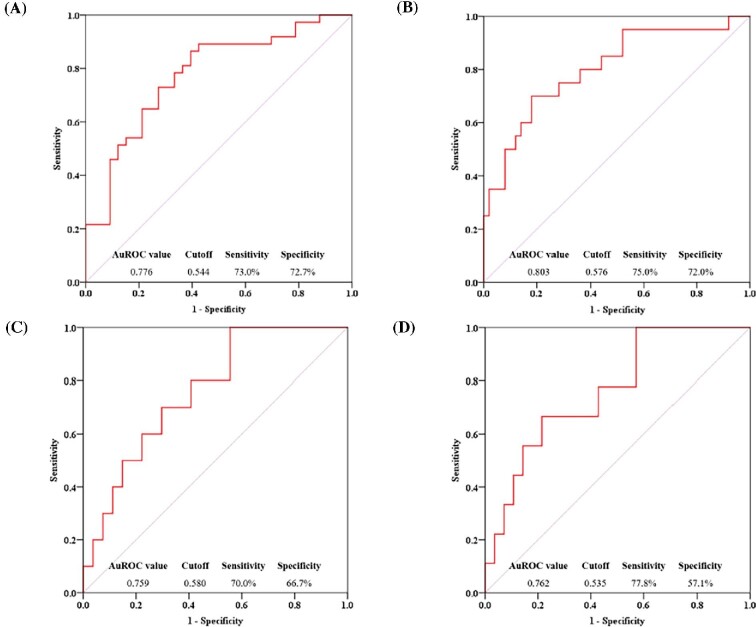
ROC curve analysis of the HRV–IDH index for predicting IDH and validation analysis of the HRV–IDH index of the second HRV test. **(A)** The AUROC of the HRV–IDH index for predicting IDH occurrence in patients who experience IDH at least once is 0.776. Based on a cut-off value of 0.544, the sensitivity is 73.0% and the specificity is 72.7%. **(B)** The AUROC of the HRV–IDH index for predicting IDH occurrence in patients who experience repeated IDH in >10% of HD sessions is 0.803. Based on a cut-off value of 0.576, the sensitivity is 75.0% and the specificity is 72.0%, thus indicating better predictive power than that for IDH occurrence in patients who experience IDH at least once. **(C, D)** To validate the usefulness of the HRV–IDH index, 12 additional HD sessions were monitored after the second HRV test. AUROC values were 0.759 and 0.762 for predicting IDH in patients who experienced IDH at least once and those who repeatedly experienced IDH, respectively, similar to the results of the HRV–IDH index of the first HRV test.

The HRV–IDH index was developed using all parameters collected from the HRV test (Supplemental Table S2): NN50, TP, VLF, LF and LF:HF ratio. The HRV–IDH index can be expressed as follows:

The developed HRV–IDH index achieved an accuracy, recall value, precision value and F1 score of 0.714, 0.784, 0.707 and 0.744, respectively. For predicting IDH in patients who experienced IDH at least once, the area under the ROC curve (AUROC) of the HRV–IDH index was 0.776. Based on a cut-off value of 0.544, the sensitivity and specificity were 73.0% and 72.7%, respectively (Fig. 2A). For predicting IDH in patients who repeatedly experienced IDH in >10% of the HD sessions, the HRV–IDH index exhibited greater predictive power—with an AUROC of 0.803—than that observed in patients who experienced IDH at least once (Fig. 2B). Moreover, IDH occurrence during 12 HD sessions after the second HRV test was further analysed using the HRV-IDH index of the second HRV test. AUROC values were 0.759 and 0.762 for predicting IDH in patients who experienced IDH at least once and those who repeatedly experienced IDH, respectively (Fig. 2C, D).

### Multivariate logistic regression for IDH occurrence and reproducibility of HRV test results

Table 4 summarizes the results of multivariate logistic regression analyses of the HRV–IDH index, factors that exhibited significant differences and known risk factors for IDH occurrence based on baseline characteristics, BCM results and TTE results. Autonomic dysfunction based on the HRV–IDH index cut-off value of 0.544 was identified as an independent risk factor for IDH (odds ratio 6.137; *P* = .011). We used G*power for post hoc power analysis, selecting logistic regression and the post hoc option to compute the achieved power. The power calculated using a two-tailed test (with α = 0.05) was 0.946.

**Table 4:** Multivariate logistic regression analysis for IDH occurrence.

Variable	Univariate OR (95% CI)	*P*-value	Multivariate OR (95% CI)	*P*-value
Autonomic dysfunction (HRV–IDH index ≥0.544)	6.210 (2.199–17.536)	.001	6.137 (1.508–24.984)	.011
Age ≥65 years	0.724 (0.281–1.868)	.505		
Female	2.875 (1.088–7.598)	.033		
DM	5.143 (1.763–15.003)	.003	3.403 (0.834–13.875)	.088
HTN	0.287 (0.082–1.005)	.051	0.203 (0.040–1.033)	.055
CHF	1.403 (0.359–5.482)	.626		
WBC count	1.531 (1.159–2.023)	.003	1.623 (1.140–2.309)	.007
Hct	1.119 (0.984–1.272)	.085		
UF rate	1.173 (0.609–2.259)	.633		
Total body water	0.930 (0.871–0.993)	.031		
Extracellular water	0.870 (0.754–1.004)	.056		
Ejection fraction	0.972 (0.923–1.023)	.279		
E:e′ ratio	0.997 (0.925–1.075)	.945	0.905 (0.812–1.008)	.070

A total of 69 patients (98.6%) were included in the multivariate logistic regression model. The *P*-value for the regression model was <.001, Negelkerke's R^2^ was 0.514 and the *P*-value of the Hosmer–Lemeshow test was .386.

CHF: congestive heart failure; CI: confidence interval; Hct: haematocrit; HTN: hypertension; OR: odds ratio; UF: ultrafiltration.

**Figure 3:**
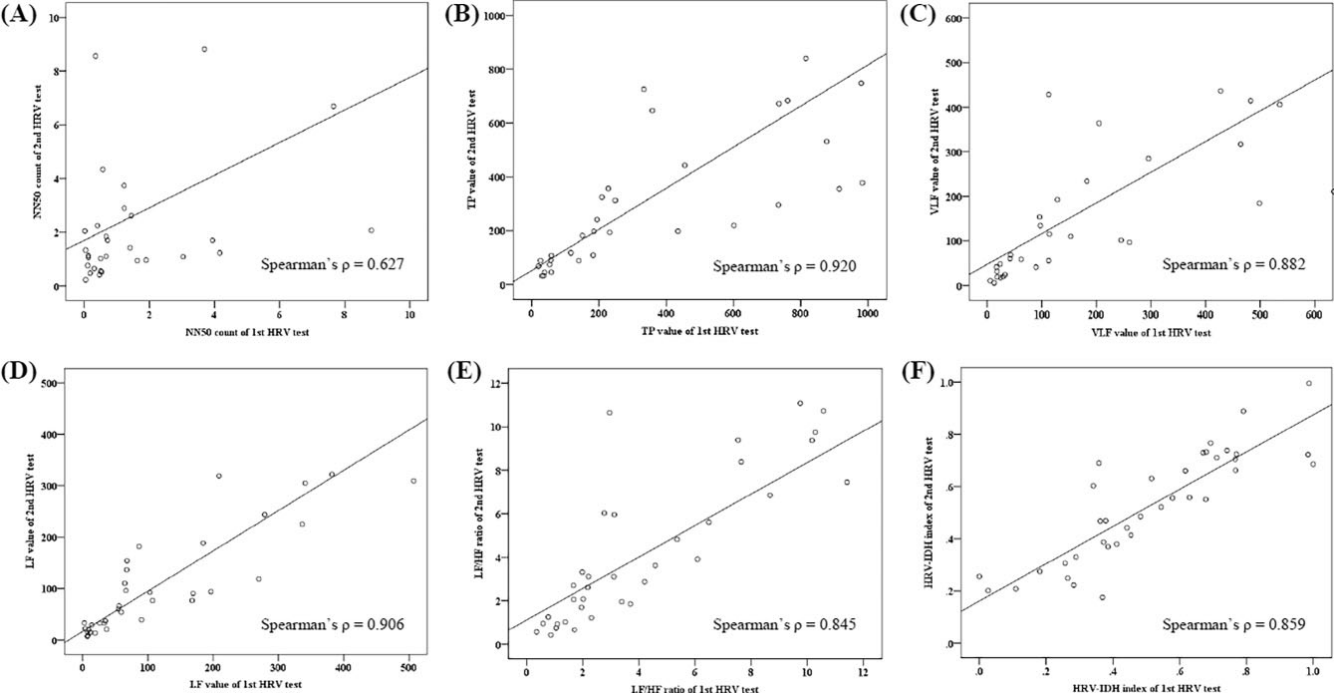
Correlation analysis of HRV parameters and HRV–IDH indices of the first and second HRV tests. **(A–E)** Spearman's ρ of all HRV parameters (NN50, TP, VLF, LF and LF:HF ratio) included in the HRV–IDH index indicate good correlations, ranging from 0.62 to 0.92. **(F)** Spearman's ρ of the HRV–IDH index is 0.859, indicating a robust correlation.

Correlation analyses to evaluate the reproducibility of HRV test results revealed strong correlations between all indices included in the HRV–IDH index (NN50, TP, VLF, LF and LF:HF ratio). Moreover, Spearman's correlation coefficient for the HRV–IDH index was 0.859, indicating a robust correlation (Fig. 3).

## DISCUSSION

We hypothesized that assessing baseline autonomic function using the HRV test would help predict IDH. The HRV–IDH index developed in this study demonstrated good predictive performance for identifying IDH. Notably, autonomic dysfunction (HRV–IDH index ≥0.544) remained an independent risk factor even after adjusting for other significant IDH factors. Additionally, duplicate HRV test parameters obtained from the same patient showed strong correlations, confirming the reliability and reproducibility of this test.

IDH occurred in 12.6% of all HD sessions observed, consistent with a previous meta-analysis reporting an 11.6% prevalence of IDH per the nadir 90 criterion [29]. Baseline characteristics evaluation indicated that the IDH group had a greater proportion of women and patients with DM, consistent with previous reports [8, 30]. The IDH group had a lower blood flow rate, dialyser surface area, incidence of renin–angiotensin system inhibitor (RASI) and calcium channel blocker (CCB) use and a greater incidence of midodrine use, all of which reflect strategies to prevent IDH [8, 31, 32]. Pre-dialysis SBP and DBP did not differ between the groups, although post-dialysis SBP and DBP were significantly lower in the IDH group. Therefore, although the IDH group exhibited stable BP at the start, it could not be maintained throughout the HD session [33].

Previous studies using the HRV test defined IDH based on the KDOQI criterion, which exhibits little correlation with patient mortality [15, 21, 22]. In contrast, our study used the nadir 90 criterion, which has the strongest association with patient mortality among the various IDH definitions [15]. Previous studies could not demonstrate the usefulness of the HRV test in predicting IDH defined by the nadir 90 criterion [21, 22]. The HRV–IDH index developed in this study included the NN50, TP, VLF, LF and LF:HF ratio of the HRV test and demonstrated good performance for predicting IDH, particularly in patients with repeated IDH. As IDH frequency is associated with poor prognosis [34, 35], the HRV–IDH index may be a valuable tool for patients with frequent IDH. Additionally, the performance of the prediction model (Youden's index = 0.451) using well-known risk factors for IDH occurrence was improved when the HRV-IDH index was added (Youden's index = 0.484), demonstrating the usefulness of the HRV test (Supplemental Table S3).

In previous studies, the HRV test was conducted during HD sessions [21, 22]. However, HD conditions, including the blood flow rate, dialyser surface area, dialysate flow rate, ultrafiltration rate and dialysate composition, may vary between sessions [36] and have a significant impact on the autonomic nervous status. Moreover, in previous studies, the LF:HF ratio was higher in the IDH group than in the non-IDH group [21, 22]. This is inconsistent with the theory that IDH occurs because sympathetic activity is not maintained until the end of HD [33] and with the results of a study showing that the LF:HF ratio during HD was lower among patients with IDH than those without [37]. Therefore, HRV tests performed during HD may not be appropriate for baseline ANF evaluation, and the reproducibility of the HRV test may be low. We used night-time HRV test results obtained during non-HD periods, which are more suitable for assessing baseline ANF. We found that the LF:HF ratio was lower in the IDH group, which is consistent with the theory that IDH is associated with lower sympathetic activity and the findings of Cavalcanti *et al*. [38]. Furthermore, the HRV test parameters (NN50, TP, VLF, LF and LF:HF ratio) and the HRV–IDH indices exhibited good correlations between the first and second HRV tests, indicating adequate reproducibility. The validation analysis of the HRV–IDH index of the second HRV test showed satisfactory results, supporting the reproducibility of the HRV test (Fig. 2).

The HRV test performed during non-HD periods can be used to predict IDH occurrence and it may also contribute significantly to IDH control by identifying patients with autonomic dysfunction among patients with IDH, thereby facilitating the use of drugs that activate sympathetic function [38–40]. We compared the 24-h HRV test results and HRV–IDH indices of healthy control, IDH and non-IDH groups using data from our previous study that analysed HRV test results between patients with end-stage kidney disease and healthy controls [18]. In healthy controls, a significantly larger HRV—i.e. intact ANF (low HRV–IDH index)—was observed, showing that the HRV test reliably determines autonomic dysfunction (Supplemental Table S4). Moreover, changes between 24-h and night-time HRV–IDH indices significantly differed only in the non-IDH group. Intra-individual daily HRV fluctuations may also warrant further research (Supplemental Table S5).

We also investigated whether the BCM and TTE results are associated with IDH occurrence. Several studies have reported the usefulness of the BCM for predicting IDH [41–43], although this still requires verification. In our study, the IDH group had a lower body water content than the non-IDH group. However, there was no intergroup difference in overhydration or oedema index. This aspect requires further investigation. Similarly, TTE usefulness in predicting IDH remains unclear [44–46], as we found no significant differences in the TTE results between the groups.

This study has some limitations. First, the HRV–IDH index model was developed using the patient population of this study, potentially limiting generalizability. The average BMI of the patients was 23.2 ± 3.7 kg/m^2^ and all patients were Korean; thus, further studies are required to determine whether the HRV test is useful in patients with obesity, underweight or of other ethnicities. Second, external validation of the HRV–IDH index model was not performed, and future validation cohort studies are needed to verify the objective usefulness of the HRV–IDH index.

In conclusion, the HRV test helps predict IDH diagnosed per the nadir 90 criterion. Specifically, it exhibits high predictive power for patients with frequent IDH and good reproducibility, which ensures test reliability.

## Supplementary Material

sfae102_Supplemental_File

## Data Availability

The data will be shared upon reasonable request to the corresponding author.
