# Supplementary material for: Usefulness of the heart rate variability test in predicting intradialytic hypotension in patients undergoing chronic haemodialysis
Source: Clin Kidney J. 2024 Apr 8;17(6):sfae102. doi: 10.1093/ckj/sfae102 (PMC11176866; doi:10.1093/ckj/sfae102)
Supplement: sfae102_Supplemental_File [file sfae102_supplemental_file.docx]

**Supplemental Table S1. Incidence of intradialytic hypotension (IDH)**

| **Total number of haemodialysis sessions** | 840 |
| --- | --- |
| **Number of IDH events (n, %)** | 106 (12.6) |
| **Patients with IDH (at least once) (n, %)** | 37 (52.9) |
| **Patients with frequent IDH (>10% of total HD sessions) (n, %)** | 20 (28.6) |

Categorical variables are expressed as numbers (percentages). HD, haemodialysis; IDH, intradialytic hypotension

**Supplemental Table S2. Development of the HRV-IDH index model for predicting intradialytic hypotension (IDH)**

|  | **B** | **SE** | ***P*-value** | **OR**  **(95% CI)** |
| --- | --- | --- | --- | --- |
| **NN50** | 0.1850 | 0.084 | 0.027 | 1.203  (1.021–1.418) |
| **TP** | -0.0344 | 0.015 | 0.025 | 0.966  (0.938–0.996) |
| **VLF** | 0.0359 | 0.016 | 0.023 | 1.037  (1.005–1.069) |
| **LF** | 0.0341 | 0.018 | 0.055 | 1.035  (0.999–1.071) |
| **LF/HF ratio** | -0.2301 | 0.125 | 0.065 | 0.794  (0.622–1.014) |
| **Constant** | 1.7394 | 0.648 | 0.007 | 5.694 |

All patients were included in the model. The *P*-value for the regression model was 0.003, Negelkerke’s R^2^ was 0.307, and the *P*-value of the Hosmer–Lemeshow test was 0.665. The accuracy was 0.714, recall value was 0.784, precision value was 0.707, and F1 score was 0.744. B, beta coefficient; CI, confidence interval; HF, high frequency; HRV, heart rate variability; IDH, intradialytic hypotension; LF, low frequency; NN50, normal to normal interval > 50 ms; OR, odds ratio; SE, standard error

**Supplemental Table S3. Comparison of the performance of the IDH prediction models with and without the HRV-IDH index**

|  | **Model 1** | **Model 2** |
| --- | --- | --- |
| **Beta coefficient** |  |  |
| Age (per 1 year) | -0.038 | -0.039 |
| Female (Ref. male) | 0.931 | 0.893 |
| DM (Ref. No) | 1.897 | 1.129 |
| HTN (Ref. No) | -1.168 | -1.302 |
| CHF (Ref. No) | 0.573 | 0.201 |
| CAD (Ref. No) | -0.541 | -1.007 |
| HRV-IDH index (per 1) | - | 4.857 |
| Constant | 1.707 | -0.042 |
| ***P*-value** | 0.004 | <0.001 |
| **Negelkerke’s R^2^** | 0.320 | 0.452 |
| **Hosmer–Lemeshow test** | 0.273 | 0.881 |
| **Accuracy** | 0.729 | 0.743 |
| **Recall value** | 0.784 | 0.784 |
| **Precision value** | 0.725 | 0.744 |
| **F1 score** | 0.753 | 0.763 |
| **AuROC value** | 0.778 | 0.839 |
| **Youden’s index^a^** | 0.451 | 0.484 |

All patients were included in both models 1 and 2. ^a^Youden's index was calculated as sensitivity + specificity - 1. AuROC, area under the receiver operating characteristics curve; CAD, coronary artery disease; CHF, congestive heart failure; DM, diabetes mellitus; HRV, heart rate variability; HTN, hypertension; IDH, intradialytic hypotension; Ref., reference

**Supplemental Table S4. Comparison of 24-hour heart rate variability test results and HRV-IDH indices among healthy controls, IDH, and non-IDH groups**

|  | Healthy controls  (n = 32) | Non-IDH  (n = 33) | IDH  (n = 37) | *P*-value |
| --- | --- | --- | --- | --- |
| SDNN (ms) | 55.0 ± 14.2^b,c^ | 28.0 ± 12.7^a^ | 21.6 ± 12.5^a^ | <0.001 |
| RMSSD (ms) | 28.1 ± 12.1^b,c^ | 14.4 ± 9.6^a^ | 13.9 ± 13.4^a^ | <0.001 |
| NN50 count | 31.03 ± 28.47^b,c^ | 7.32 ± 17.27^a^ | 9.44 ± 20.62^a^ | <0.001 |
| pNN50 (%) | 10.63 ± 10.64^b,c^ | 2.68 ± 6.79^a^ | 3.43 ± 8.65^a^ | 0.001 |
| Total power (ms^2^) | 1751.5 ± 899.0^b,c^ | 442.8 ± 441.9^a^ | 274.0 ± 351.0^a^ | <0.001 |
| VLF (ms^2^) | 860.6 ± 407.4^b,c^ | 221.8 ± 246.8^a^ | 123.0 ± 161.3^a^ | <0.001 |
| LF (ms^2^) | 607.4 ± 319.4^b,c^ | 146.1 ± 167.3^a^ | 84.3 ± 139.6^a^ | <0.001 |
| HF (ms^2^) | 257.5 ± 219.7^b,c^ | 58.6 ± 89.2^a^ | 45.7 ± 76.1^a^ | <0.001 |
| LF/HF ratio | 4.46 ± 1.82^b,c^ | 4.68 ± 3.41^a^ | 3.11 ± 2.51^a^ | 0.031 |
| **HRV-IDH index** | 0.25 ± 0.22^b,c^ | 0.47 ± 0.22^a,c^ | 0.63 ± 0.21^a,b^ | <0.001 |

Continuous variables are expressed as mean ± standard deviation. ^a^P < 0.017 versus healthy controls, ^b^P < 0.017 versus the non-IDH group, ^c^P < 0.017 versus the IDH group. HF, high frequency; HRV, heart rate variability; IDH, intradialytic hypotension; LF, low frequency; NN50, number of pairs of adjacent normal to normal intervals differing by >50 ms; pNN50, percentage of NN50 count; RMSSD, root mean square of successive normal to normal intervals; SDNN, standard deviation of normal to normal interval; VLF, very low frequency

**Supplemental Table S5. Comparison of 24-hour and nighttime HRV-IDH indices in IDH and non-IDH groups**

|  | 24-hour HRV-IDH index | Nighttime HRV-IDH index | *P*-value |
| --- | --- | --- | --- |
| **Non-IDH group** | 0.47 ± 0.22 | 0.41 ± 0.22 | 0.004 |
| **IDH group** | 0.63 ± 0.21 | 0.63 ± 0.21 | 0.940 |

Continuous variables are expressed as mean ± standard deviation. HRV, heart rate variability; IDH, intradialytic hypotension

**Supplemental Figure S1. Study population**


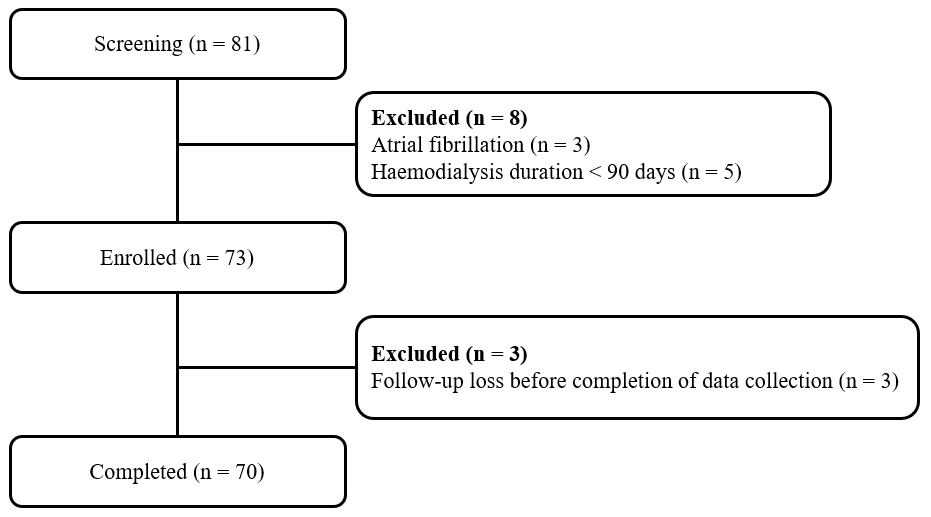


Among the 81 screened patients, 3 with atrial fibrillation and 5 with a haemodialysis vintage of <90 days were excluded. Three patients were lost to follow-up during the study period. Complete data were collected for 70 patients.
